# Attentional influences on neural processing of biological motion in typically developing children and those on the autism spectrum

**DOI:** 10.1186/s13229-022-00512-7

**Published:** 2022-07-18

**Authors:** Emily J. Knight, Aaron I. Krakowski, Edward G. Freedman, John S. Butler, Sophie Molholm, John J. Foxe

**Affiliations:** 1 The Frederick J. and Marion A. Schindler Cognitive Neurophysiology Laboratory, Department of Neuroscience, University of Rochester School of Medicine and Dentistry, The Del Monte Institute for Neuroscience, University of Rochester Medical Center, 601 Elmwood Avenue, Box 603, Rochester, NY 14642 USA; 2 Division of Developmental and Behavioral Pediatrics, Department of Pediatrics, University of Rochester Medical Center, School of Medicine and Dentistry, University of Rochester, 601 Elmwood Avenue, Box 671, Rochester, NY 14642 USA; 3 The Cognitive Neurophysiology Laboratory, Department of Pediatrics and Neuroscience, Albert Einstein College of Medicine, Bronx, NY USA; 4 Program in Cognitive Neuroscience, The Graduate Center of the City University of New York, 365 Fifth Avenue, New York, NY 10016 USA; 5 School of Mathematical Sciences, Technological University Dublin, Kevin Street, Dublin, Ireland

**Keywords:** ASD, Event-related potentials, ERP, Social cognition, Visual evoked potential, VEP, Biological motion

## Abstract

**Background:**

Biological motion imparts rich information related to the movement, actions, intentions and affective state of others, which can provide foundational support for various aspects of social cognition and behavior. Given that atypical social communication and cognition are hallmark symptoms of autism spectrum disorder (ASD), many have theorized that a potential source of this deficit may lie in dysfunctional neural mechanisms of biological motion processing. Synthesis of existing literature provides some support for biological motion processing deficits in autism spectrum disorder, although high study heterogeneity and inconsistent findings complicate interpretation. Here, we attempted to reconcile some of this residual controversy by investigating a possible modulating role for attention in biological motion processing in ASD.

**Methods:**

We employed high-density electroencephalographic recordings while participants observed point-light displays of upright, inverted and scrambled biological motion under two task conditions to explore spatiotemporal dynamics of intentional and unintentional biological motion processing in children and adolescents with ASD (*n* = 27), comparing them to a control cohort of neurotypical (NT) participants (*n* = 35).

**Results:**

Behaviorally, ASD participants were able to discriminate biological motion with similar accuracy to NT controls. However, electrophysiologic investigation revealed reduced automatic selective processing of upright biologic versus scrambled motion stimuli in ASD relative to NT individuals, which was ameliorated when task demands required explicit attention to biological motion. Additionally, we observed distinctive patterns of covariance between visual potentials evoked by biological motion and functional social ability, such that Vineland Adaptive Behavior Scale-Socialization domain scores were differentially associated with biological motion processing in the N1 period in the ASD but not the NT group.

**Limitations:**

The cross-sectional design of this study does not allow us to definitively answer the question of whether developmental differences in attention to biological motion *cause* disruption in social communication, and the sample was limited to children with average or above cognitive ability.

**Conclusions:**

Together, these data suggest that individuals with ASD are able to discriminate, with explicit attention, biological from non-biological motion but demonstrate diminished automatic neural specificity for biological motion processing, which may have cascading implications for the development of higher-order social cognition.

**Supplementary Information:**

The online version contains supplementary material available at 10.1186/s13229-022-00512-7.

## Introduction

Social processing is a hallmark of human cognition and socially salient signals can be detected from greatly impoverished sensory information. A prime example is the highly specialized ability to detect diverse social information, such as emotion, mood, and gender from biological motion point-light displays, in which body movements are represented through a small set of dots attached to the joints of humans as they perform common motions [1–4].

Remarkably, preference for upright biological motion over scrambled or inverted motion point-light displays already appears to be present in children as young as 2 days old [5, 6] and evolves rapidly over the first 2 years of life [7]. This preference for upright motion suggests an innate predisposition to biological motion perception, with obvious implications for preferential detection of and responding to caretakers early in life [5]. Furthermore, accuracy in recognizing human and non-human biological motion increases from ages 3 to 5 years, indicating that efficiency of biological motion processing continues to develop across early childhood in typical development [8]. Given the obvious link between biological motion processing and the ability of an observer to decode the emotional state or intentions of another, it is not surprising that researchers have been interested in determining whether this capability is weakened in individuals with autism spectrum disorder (ASD) [9], a neurodevelopmental disorder characterized by difficulties in social interaction and communication [10].

Unfortunately, the literature has produced somewhat mixed results to date. The earliest behavioral findings from Moore and colleagues reported a significant impairment in children and adolescents with ASD in the inference of internal emotional and mental states from biological motion point-light displays, but not in the categorization of overt biological actions (e.g., running, jumping, etc.) [11]. This finding, which has been replicated in studies of high-functioning children with ASD as well as adolescents and young adults with ASD, is consistent with an emotion-processing dysfunction rather than a more fundamental sensory–perceptual biological motion processing disorder [12, 13]. Likewise, a lack of observed differences in biological motion identification and perception of specific biological motion features (i.e., kinematic profile, action discrimination in noise, direction discrimination in noise) provide further evidence for intact basic processing of biological motion in children, adolescents, and adults with ASD [14–19]. Yet, other studies do report significant differences in recognition of biological motion, or discrimination of actions or direction of biological motion among those with ASD [20, 21]. Atkinson and colleagues also describe a correlation between impaired emotion detection and motion coherence thresholds in adults with ASD, suggesting that emotion detection impairments may be partially explained by a lower-level visual motion processing deficit [22]. Furthermore, associations between reduced biological motion processing and clinical autism symptom severity, as measured by the Autism Diagnostic Observation Schedule and Childhood Autism Rating Scale or Autism Quotient, have been described [23, 24], affording some additional support for the notion that disruption of biological motion processing may be important in ASD. Three meta-analyses have attempted to integrate findings from these individual studies exploring the perception of biological motion in point-light displays [25–27]. These meta-analyses reveal an emerging pattern of overall small-to-moderate deficits in biological motion perception in ASD, but confirm high heterogeneity among studies. They also confirm that deficits in biological motion processing among autistic individuals tend to be more severe when inferring social implications including intentionality or emotion from biological motion, as opposed to lower-level detection of biological motion and discrimination of action or direction of biological motion in noise.

Likewise, neuroimaging studies in general suggest that individuals with ASD have biological motion task-related hypoactivation in brain regions previously implicated in biological motion processing. This includes the right superior temporal region [19, 28–31], an area that also appears highly linked to social cognition in ASD [32, 33], as well as altered activation in right middle and inferior temporal gyri, middle frontal gyrus, inferior parietal lobule, and supplementary motor area [26, 30, 34]. Importantly, altered brain activation has been observed regardless of the presence of identifiable performance deficits, suggesting that individuals with ASD may employ different brain networks to accomplish the same goal of biological motion processing [19, 30]. Additionally, specific patterns of neural activation during biological motion processing have been linked with clinical symptoms in ASD. For example, hypoactivation of the superior temporal sulcus (STS), inferior frontal gyrus and cerebellum during biological motion processing have been linked with higher ASD symptom severity [35, 36] and impaired gesture production [37], and pre-treatment STS activation selectivity to biological over scrambled motion predicts improvement in ASD symptoms with pivotal response therapy [38]. Furthermore, STS activation during biological motion processing is one functional region that can distinguish aspects of familial risk for ASD [39, 40]. While the STS has repeatedly been implicated in fMRI studies of biological motion processing in ASD, our understanding of associated temporal dynamics is somewhat more limited as fewer electrophysiologic studies have been conducted to date. Studies of oscillatory dynamics including mu suppression and beta activity during passive observation of biological motion have revealed no differences between individuals with ASD and controls [26, 41, 42]. When examining evoked potentials to biological motion, Kroger and colleagues have found group differences in P1 amplitude along with atypical lateralization of visual evoked potentials during processing of both biological and scrambled motion in ASD. This suggests deficits in general visual motion processing or processing of complex stimuli rather than a biological motion-specific deficit [43]. However, a trend toward decreased right hemisphere P1 in response to biological motion has been observed following social skills training in individuals with ASD, paralleling the findings of the neuroimaging literature by drawing a potential link between electrophysiologic mechanisms of biological motion processing and autism symptomatology [44].

Thus, although emerging evidence highlights possible differences in neural mechanisms of biological motion processing in ASD, as well as potential implications for social function, a high degree of inconsistency in findings makes it difficult to draw definitive conclusions. Variability in findings cannot be accounted for by within-subject unreliability as several studies have shown that behavioral and electrophysiologic responses to visual paradigms are highly consistent within individuals on repeated measures [45, 46]. Instead, the high study heterogeneity is more likely to reflect the high variability inherent in the phenotypes of individuals with autism spectrum disorder. Given this high degree of interindividual phenotypic difference, study populations are likely to vary across studies both on commonly measured dimensions such as age, IQ and verbal abilities, as well as on areas that are not as extensively characterized. Some key potential mediators of variability that are important to consider in biological motion processing include but are not limited to between-subjects developmental, cognitive and/or attentional factors. There are clear maturational changes in biological motion processing, and developmental trajectories appear to differ in ASD as compared to typical development. While NT children from ages 5–12 years steadily improve in their perceptual sensitivity to biological motion, children with ASD show a flat developmental trajectory, overlapping with NTs only at the youngest age, pointing to atypical development of biological motion perception in ASD [20]. Although impaired recognition of biological motion has also been observed in adults with ASD [47], looking across studies, it appears that differences between ASD and NT participants decrease with age, as children with ASD show more substantial deficits than adolescents and adults [26], and the majority of studies of older adolescents and adults report no differences in behavioral performance on biological motion tasks.

The association between cognitive factors and biological motion processing has also been explored as a source of heterogeneity, but does not appear to be strongly influential in accounting for variability in study results. Individual studies have suggested that IQ may correlate with some aspects of biological motion processing ability only among those with ASD [15–18, 21]. However, meta-analyses suggest minimal overall influence of IQ on biological motion processing [25, 26].

In contrast, attention remains a strong candidate mediator for some of the inconsistency in findings across studies and biological motion paradigms, since studies that have demonstrated correlation between differential fixations and task performance in biological motion paradigms suggest a modulatory role of attention [41, 48, 49]. Additionally, a recent fMRI study showed that, unlike NT adults, adults with ASD did not have augmentation of STS functional activity in an explicit biological motion processing task compared to passive processing task [50], suggesting an inability to adapt to attentional task demands.

It is possible that this dysfunction results from very early attentional differences in children with ASD. There seems to be a fundamental difference in preferential attendance to biological motion in children with ASD in passive viewing paradigms as, unlike NT preschoolers, 2–3 years olds with ASD did not show a preference for upright over inverted motion in point-light displays or for motion of people over geometric shapes, and school-age children and adolescents with ASD likewise do not show a preference for biological over non-biological motion point-light displays [51–54]. In fact, patterns of fixation to biological over non-biological motion can even help distinguish children with ASD from NT children [55] or predict a future diagnosis of ASD in preschoolers [53]. Even older adolescents and adults with ASD show a lower percentage of time fixating on movies of real people than on geometric shapes; however, they do not show a difference from NT controls in preferential fixation on upright vs. inverted motion point-light displays [56]. This may be due to developmental changes in biological motion processing, or may relate to the complexity of social stimuli [51, 52, 56, 57]. It is important to note that large individual variability in biological motion preference (range = 11.6–90.2%) has also been reported in 3-year-old children with ASD, such that 8 out of 20 did show an intact biological motion preference [53]. As biological motion preference also covaries with adaptive functioning and predicted reduction in symptom severity a year later, attendance to biological motion may well prove a sensitive measure of behavioral characteristics in children with ASD and have a cascading influence on developmental trajectory [53].

Therefore, in parsing the heterogeneity of findings in the biological motion literature it is reasonable to suggest that although individuals with ASD appear largely capable of discriminating basic non-emotional features of biological motion when explicitly directed to do so, they may differentially attend to biological motion which could impact detection of relevant social cues. As the modulatory role of attention on neural mechanisms of low-level biological motion processing in ASD remains incompletely understood, the current study takes advantage of a behavioral and electrophysiologic paradigm previously developed by our research group [58] to map spatiotemporal dynamics of unattended and explicitly attended processing of biological motion point-light displays in typical development and ASD.

## Methods

### Participants

Thirty-two children and adolescents with ASD and thirty-seven age-matched neurotypical children (NT) aged 6–16 years were initially enrolled in the study. Two controls and five children with ASD were excluded from the analysis as a result of failure to meet minimum accuracy criterion on the behavioral performance (see “Measurements and Analyses: Behavioral Analyses”), and six NT and four ASD children were excluded for missing age and IQ data. Therefore, final primary analyses were conducted on 22 ASD and 31 NT children. Participant characteristics are outlined in Table 1.

**Table 1:** Participant characteristics

	NT (*n* = 31)	ASD (*n* = 22)	Significance
Mean age (SD, range)	11.6 (3.0, 6–16)	10.6 (2.6, 7–15)	*p* = *.212*
Mean IQ (SD)	114 (14.5)	107 (20)	*p* = *.173*
Percentage male	46%	74%	*p* = *.025*

Participants were screened for normal or corrected-to-normal vision in both eyes. Exclusion criteria for both groups included a history of seizures or head trauma. Other exclusion criteria for the NT group included a history of developmental, psychiatric, or learning difficulties as assessed by a parent history questionnaire. NT children were also excluded if they had a biological first-degree relative with a known developmental disorder. All children were screened for attention deficit hyperactivity disorder (ADHD). Only NT children were excluded if their parents endorsed six items or more of inattention or hyperactivity on a DSM-IV ADHD behavioral checklist. Children with ASD were not excluded for presenting with symptoms of inattention and hyperactivity, as such symptoms are very common in ASD. However, only one participant in the ASD group endorsed history of clinician-documented ADHD. Parents of ASD participants on stimulant medication were asked to refrain from administering the stimulant medication during the 24-h period prior to electrophysiological testing.

A diagnosis of autism was validated through the Autism Diagnostic Interview-Revised [59], the Autism Diagnostic Observational Schedule [60] and judgment by a licensed clinician. All children were administered the Wechsler Abbreviated Scale of Intelligence [61] for the purposes of characterizing and matching the samples. Exclusion criteria for both the groups included a Full Scale IQ below 70 as assessed by the Wechsler Abbreviated Scale of Intelligence. Functional social communication was assessed for all participants using the Vineland Adaptive Behavior Scale-2nd Edition [62].

The parent or guardian of each child provided written informed consent, and developmentally appropriate assent was obtained from the child. All procedures had prior approval by the institutional review boards of the Albert Einstein College of Medicine and the City University of New York. Participants received modest remuneration for their time.

### Stimuli and tasks

**Fig. 1:**
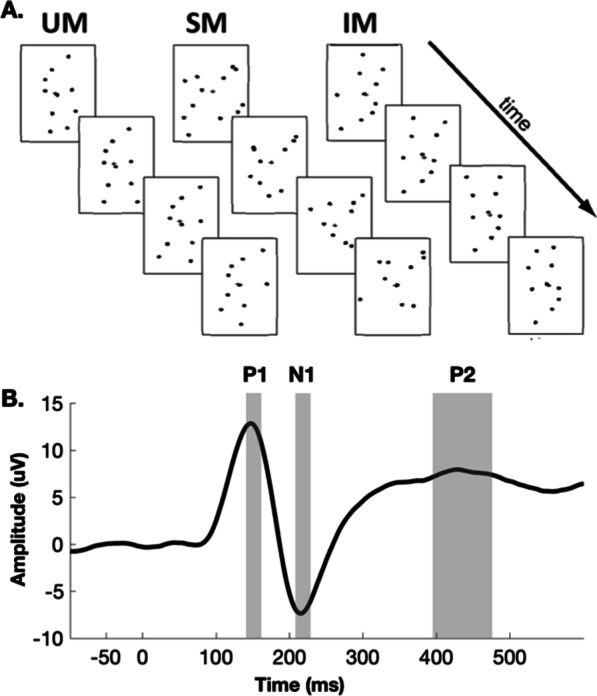
**A.** Sequences of frames from sample clips of upright biological motion (UM), inverted biological motion (IM) and scrambled biological motion (SM). **B.** Components used in the stage one analysis as overlaid over the two analyzed bilateral parieto-occipital sites (PO7 and PO8), collapsed across all groups (ASD and TDs), conditions (UM, SM and IM) and tasks (unattended and attended)

Video clips of an adult human engaged in common activities (e.g., running, kicking, climbing, throwing and jumping) were imported to a computer to create three stimulus types: upright biological motion (UM), scrambled motion (SM) and inverted biological motion (IM) stimuli (Fig. 1a). Markers were placed on the actor’s joints in each frame of the sequence, such that the final clips were only composed of up to twelve moving dots (i.e., point-light displays). SM sequences were created from the normal biological animations and consisted of the same individual dots undergoing the same local motions as the biological counterparts. Scrambling was achieved by randomizing the spatial locations of the dots in a given animation, thereby distorting the hierarchical, pendular motions that are characteristic of biological motion. As such, SM only retains intact local motion information that is insufficient for the perception of a human actor, thus establishing a control stimulus for undistorted UM. IM sequences were created from the normal, UM by rotating the images 180 degrees. IM retains the overall global configuration of the point-light displays and is generally detectable as biological motion (at least by adults). Nonetheless, IM stimuli are not as readily interpretable as UM [5, 63, 64]. The methodology behind the generation of biological motion sequences is discussed more fully in previous studies [23, 65].

Displays were presented on a 26″ ViewSonic® VP2655wb monitor controlled by Neurobehavioral Systems™ Presentation® software. All experiments were conducted in a sound-attenuated electrically shielded room illuminated by light from the video screen. Participants were instructed to maintain fixation on a central fixation cross and eye position was monitored by vertical and horizontal electrooculogram.

Participants performed two tasks, henceforth termed “unattended” and “attended.” For each task, they were presented with six five-minute blocks of randomly ordered video clips of all three stimulus types (UM, SM, and IM). The unattended task targeted involuntary, automatic processing of biological motion while participants performed a distractor task. In all clips, one of the dots briefly (67-ms duration) turned either red or green and participants were instructed to respond to the color change by depressing one of two mouse keys in a two-alternative forced choice task. To ensure that attention was maintained throughout the duration of the clip to the entirety of the display, without reliance on prior experience, this color change occurred at both random time and location. To maintain presumed naïveté in the unattended task, participants were not explicitly informed that some of the clips portrayed human motion until the attended task, which always followed completion of the entire unattended task. As such, the electrophysiological differences in response to the different stimuli could be said to reflect primarily unintentional processing of biological motion.

In the attended task, participants were presented with the same video clips. However, instead, participants were asked to judge whether or not the clips depicted human motion by depressing one of two mouse keys (i.e., “the dots move like a person” [both “upright" and “upside-down”] vs. they “don’t look like a person”). As such, the second task explored intentional processing of biological motion. The exact same stimuli, including the color-changing dot, were used in both tasks to ensure that differences found in the responses to the two task conditions were clearly reflective only of the actual task manipulation difference. In both tasks, subjects were also instructed to delay their responses until the completion of each video clip in order to diminish the impact of motor response-related artifacts. As a result, behavioral measures included accuracy but not reaction time.

In total, 120 distinct video clips were used, 40 of which represented upright point-light displays of canonical biological motion, 40 of which represented inverted point-light displays of canonical biological motion, and 40 of which represented scrambled motion. Stimuli appeared as black against a white background and consisted of approximately twelve dots (variable depending on the motion represented by the figure) moving within 5.8° of visual angle. Each clip was composed of 29 frames presented at the monitor refresh rate of 60 Hz, for a total frame duration of 17 ms and a total clip duration of 483 ms. Because the practical constraints involved in studying young and/or clinical populations necessitated relatively brief recording sessions, the inter-stimulus interval was also kept relatively short (500-1000 ms) to ensure that sufficient electrophysiological data could be obtained for analysis. Both tasks were preceded by a set of practice trials to ensure participants understood the task. Over the course of both tasks, participants were encouraged to take breaks between blocks as necessary in order to maintain high concentration and reduce fatigue.

### Measurements and analyses

#### Behavioral analysis

D-prime scores were calculated for correctly identifying biological motion (UM, IM) as compared to SM in the attended condition and for correctly identifying green as compared to red color change in the unattended condition. In signal detection theory, D-prime serves as an index of the actual signal relative to the noise and can be measured as the difference between the normalized hit rates and false alarm rates [66]. Participant data were excluded from all subsequent analyses if the average d-prime score across the attended and unattended conditions was less 0.25, resulting in the exclusion of 5 ASD and 2 NT participants. Ten participants (4 ASD, 6 NT) were missing age and/or IQ data. Participants with missing data were also excluded. Performance in the resulting two groups (n_ASD_ = 22, n_NT_ = 31) in each of the two conditions was compared using a generalized linear model with covariates of IQ and age. For all analyses, alpha criterion was set at 0.05.

#### Electrophysiology

A BioSemi ActiveTwo (BioSemi B.V., Amsterdam, Netherlands) 64-electrode array was used to record continuous EEG signals. The setup includes an analog-to-digital converter and fiber-optic pass-through to a dedicated acquisition computer (digitized at 512 Hz; DC- to-150 Hz pass-band). Data were referenced to an active common mode sense electrode and a passive driven right leg electrode. EEG data were processed and analyzed offline using custom scripts that included functions from the EEGLAB [67] and ERPLAB Toolboxes [68] for MATLAB [69]. After acquisition, data were re-referenced to a medial frontal site (FPz) for analysis and filtered using an IIR Butterworth filter (roll-off 12db/oct, 40db/dec, order 2) with a bandpass set at 0.1–40 Hz. Bad channels were automatically or manually rejected and then interpolated using EEGLAB spherical interpolation. Data were then divided into epochs that started 100 ms before the presentation of each stimulus and extended to 1300 ms post-stimulus onset. All epochs were baseline-corrected to the 50-ms pre-stimulus interval. Trials containing severe movement artifacts or particularly noisy events were rejected if voltages exceeded ± 125 μV. Because of the relatively long duration of each video stimulus, artifacts were only rejected before 600 ms. The number of interpolated channels and accepted trials for each condition and group is presented in Additional file 1. The remaining artifact-free epochs were averaged separately for each condition, and to isolate biological motion-specific processing, we also obtained the difference in evoked response between UM and SM.

In order to examine the effects of biological motion processing and attention in ASD and NT, while limiting type-II errors, the initial analysis was restricted both spatially and temporally. A pair of bilateral regions-of-interest comprising electrode sites on or near the parieto-occipital junctions bilaterally (PO7 and PO8) were defined based on our findings in NT adults using a near-identical paradigm to the present study, in which we sourced biological motion-related activity to underlying higher-order visual processing areas such as posterior STS [58]. Mean amplitudes were computed for visual evoked potential components for each participant unbiased by morphology of individual waveforms. The latency windows for each component were first broadly defined based on component latency windows that were identified in a previous study mapping the spatiotemporal dynamics of biological motion processing using this paradigm in neurotypical adults [58]. Then, appreciating the different age range of participants in this study, peak latency was further refined within these general component time windows based on grand-averaged waveforms collapsed across all groups, tasks, and stimuli (i.e., without regard for or bias from the dependent measures of interest). These encompassed three time windows centered at peak activity (P1 = 136–156 ms; N1 = 207–227 ms; P2 = 370–450 ms) (Fig. 1b). As the earliest components were more “dynamic” with higher frequencies, their duration is correspondingly shorter than the later, lower frequency P2 component. For each of these components, we first implemented in SPSS (IBM Corp. Released 2020. IBM SPSS Statistics for macOS, Version 27.0. Armonk, NY: IBM Corp) separate generalized linear mixed model analyses (i.e., mixed design analyses of variance) with a between-subjects factor of group (NT, ASD) and within-subject factors of motion type (UM, IM, SM), task (unattended, attended), and hemisphere (left-PO7, right-PO8), with age and IQ as covariates. As the stage 1 electrophysiologic analysis consisted of three planned analyses of variance to test specific hypotheses related to the a priori defined components, uncorrected *p* values are reported.

Given the skewed sex ratio in the ASD group, we also explored any effect of sex by conducting a post hoc analysis separately on the NT group only. Data from NT participants were analyzed using a generalized linear model with a between-groups factor of sex and within-subject factors of motion type (UM, SM, IM), task (unattended, attended), and hemisphere (right, left), as well as age and IQ as continuous covariates across the same three P1, N1, and P2 time windows used in the primary analysis.

Finally, in order to incorporate more fully the wealth of information provided by our high-density electrophysiological dataset, we also implemented an exploratory factorial mass analysis across all 64 scalp electrodes and time points (100–500 ms) in a 2 × 3x2 factorial design with a between-subjects factor of group (NT, ASD) and within-subject factors of motion type (UM, IM, SM) and task (unattended, attended). We controlled family-wise error via a cluster-corrected mass permutation approach, which presumes that true evoked effects are more likely than noise to be co-located in space and time [70, 71]. Given that age and IQ were not used as covariates in these exploratory analyses, all data that met minimum behavioral accuracy criteria, including those participants missing age or IQ data, were included (n_NT_ = 35, n_ASD_ = 27). We used a family-wise alpha level of 0.05 and default 10,000 permutations. Any electrodes within approximately 5.44 cm of one another were considered spatial neighbors. Data points that are spatially and temporally adjacent and that exceed the threshold for inclusion are considered a cluster. All *F* values in the cluster are then summed, and compared to a null distribution for cluster mass significance estimated with permutations. This analysis was implemented in the Factorial Mass Univariate Toolbox [72], which extends the existing Mass Univariate Toolbox [70]. The approach to calculation of effects in multifactorial designs is based on prior simulation work [73] and detailed documentation is provided by the creators of the Factorial Mass Univariate Toolbox (https://github.com/ericcfields/FMUT/wiki/Mass-univariate-statistics-and-corrections). Briefly per this documentation, the Factorial Mass Univariate Toolbox first reduces the data to the simplest design that can calculate an equivalent test. Then, for all effects here not involving diagnostic group the method of restricted permutation would be used such that permutations occur only within-subjects with the between-subjects effect held constant. For interaction effects here that involved diagnostic group, the method would then default to permutation of residuals [73]. These permutation methodologies produce effect estimates that, while approximations, maintain an acceptable Type I error rate approaching *α* = 0.05, and are therefore appropriate for the exploratory analysis in this study.

### Correlations of biological motion processing with social ability

To assess the relationship between biological motion processing and functional social ability, we conducted multiple regression analyses separately within each diagnostic group, correlating Vineland Adaptive Behavior Scale-2nd Edition Socialization domain scores with the difference in mean amplitude between the UM and SM waveforms in the unattended and attended tasks (averaged across the PO7 and PO8 electrode sites) within each of the three-time windows of interest (P1, N1, P2). Participants for whom a Vineland was not completed (6 ASD, 10 NT) were excluded from this analysis.

## Results

### Behavioral performance

There was no statistically significant main effect of diagnostic group on performance as measured by d-prime (F(1,49) = 0.670, *p* = 0.417, η_p_^2^ = 0.013) nor main effect of task (F(1,49) = 0.657, *p* = 0.421, η_p_^2^ = 0.013), and there was no group x task interaction (F(1,49) = 0.901, *p* = 0.347, η_p_^2^ = 0.018) after controlling for age and IQ. However, on average the ASD group did appear to perform slightly more poorly than the NT group, particularly in the attended condition (Unattended: $\overline{x }$_ASD_ = 1.31 ± 0.81; $\overline{x }$_NT_ = 1.51 ± 0.72; Attended: $\overline{x }$_ASD_ = 1.07 ± 0.79; $\overline{x }$_NT_ = 1.50 ± 0.78). D-prime values for all groups and conditions are presented in Additional file 2. The lack of task main effect indicates that the tasks did not significantly differ in difficulty. There were main effects of both age (F(1,49) = 5.536, *p* = 0.023, η_p_^2^ = 0.102) and IQ (F(1,49) = 10.921, *p* = 0.002, η_p_^2^ = 0.182), with older or higher IQ participants tending to demonstrate better performance (see Additional file 3).

### Electrophysiology

#### Stage one analysis: regions-of-interest and ERP components

**Fig. 2:**
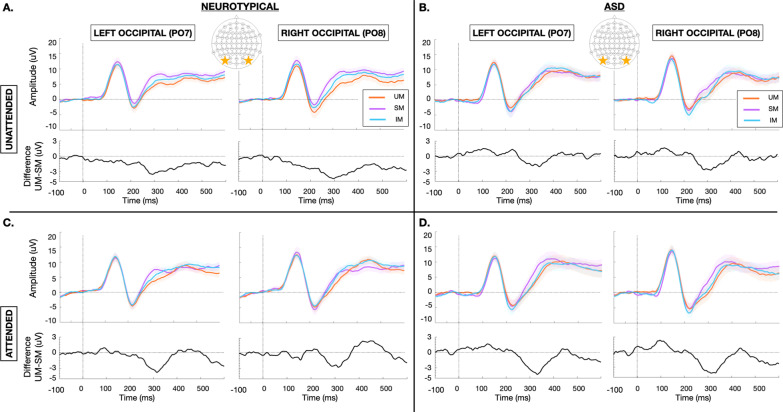
Grand average visual evoked potentials at bilateral parieto-occipital sites (PO7 and PO8) obtained in NT (**A**, **C**) and ASD (**B**, **D**) participants to UM (orange), SM (purple), and IM (blue) in the unattended (**A**, **B**) and attended (**C**, **D**) tasks. Shaded regions mark ± SEM. Black tracing represents the difference waveform of the evoked potential to UM minus SM

Grand-average visual evoked potentials at the a-priori defined regions of interest (PO7 and PO8) for each of the three types of motion (UM, SM, IM) in the attended and unattended tasks are presented for each group (NT, ASD) in Fig. 2. To highlight biological motion-specific processing, the difference in evoked response to UM minus SM is also represented for each group and attentional task. To aid in visualization of age-related effects, grand average evoked potentials to each motion type subdivided by younger (age 6–10) and older (age 11–16) participants are presented in Additional file 4. In the initial analysis, data were analyzed using separate generalized linear mixed model designs for each of the three time windows of interest. Results of this stage one analysis are summarized in Table 2.

**Table 2:** Stage 1 analysis results summary

		P1 (136-156 ms)	N1 (207-227 ms)	P2 (370-450 ms)
		F (*p* value)		
**Main effects**				
Group	df (1,49)	.209 (*p* = .649)	.000 (*p* = .992)	.239 (*p* = .627)
Hemisphere	df (1,49)	.202 (*p* = .655)	4.061 (*p* = .049)*	.718 (*p* = .401)
Task	df (1,49)	2.069 (*p* = .157)	.208 (*p* = .650)	.007 (*p* = .934)
Motion-type	df (2,98)	.716 (*p* = .491)	.360 (*p* = .699)	2.262 (*p* = .110)
**Within subjects interactions**				
Hemisphere x Task	df (1,49)	.292 (*p* = .592)	.558 (*p* = .458)	.385 (*p* = .538)
Hemisphere x Motion-type	df (2,98)	1.088 (*p* = .331^a^)	.297 (*p* = .717^a^)	2.023 (*p* = .147^a^)
Task x Motion-type	df (2,98)	.578(*p* = .563)	2.566 (*p* = .094)	.057 (*p* = .945)
Hemisphere x Task x Motion-type	df (2,98)	.217 (*p* = .756^a^)	.471 (*p* = .606^a^)	.178 (*p* = .762^a^)
**Within subject x between subject interactions**				
Hemisphere x Group	df (1,49)	.693 (*p* = .409)	.001 (*p* = .979)	3.622 (*p* = .063)
Task x Group	df (1,49)	.063 (*p* = .803)	.227 (*p* = .636)	.003 (*p* = .856)
Motion-type x Group	df (2,98)	3.108 (*p* = .049)*	2.944 (*p* = .057)	.552 (*p* = .568)
Hemisphere x Task x Group	df (1,49)	.961 (*p* = .386)	.347 (*p* = .558)	.040 (*p* = .842)
Hemisphere x Motion-type x Group	df (2,98)	.934 (*p* = .380^a^)	.826 (*p* = .429^a^)	.039 (*p* = .938^a^)
Task x Motion-type x Group	df (2,98)	.961 (*p* = .386)	**4.780 (*p* = .016**^**a**^)*	6.818 (*p* = .002)**
Hemisphere x Task x Motion-type x Group	df (2,98)	.752 (*p* = .448^a^)	1.226 (*p* = .298^a^)	.403 (*p* = .600^a^)
**Covariates**				
Age	df (1,49)	14.874 (*p* < .001)**	1.270 (*p* = .265)	4.169 (*p* = .047)*
IQ	df (1,49)	.520 (*p* = .649)	.009 (*p* = .923)	.087 (*p* = .769)

Bold values indicate significant results (p < 0.05)

Degrees of freedom (df) reported as uncorrected (sphericity assumed) values though where appropriate *p* values marked as reflecting

n_NT_ = 31,n_ASD_ = 22

^a^Greenhouse–Geisser correction for violation of Mauchly’s test of sphericity

*p < .05, **p < .01

**Fig. 3:**
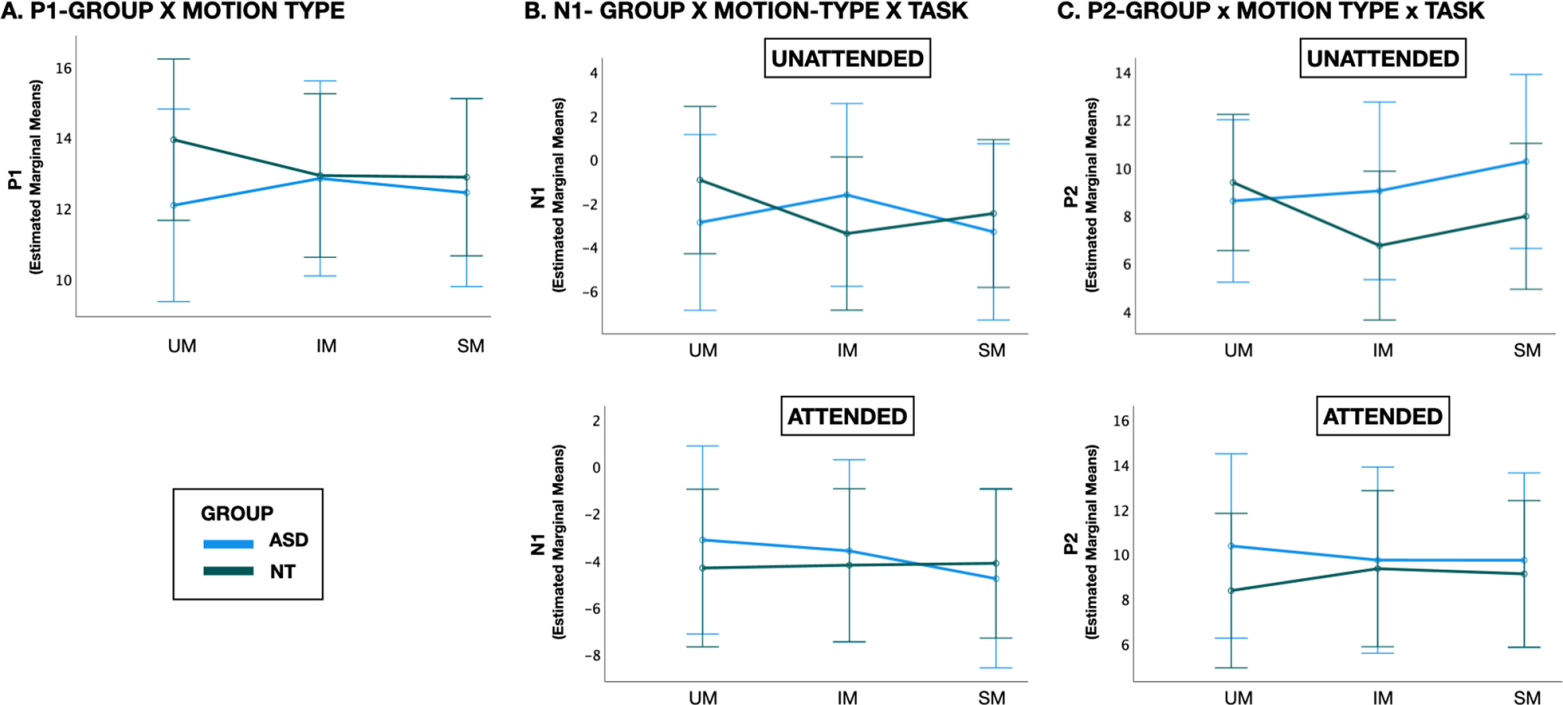
Mean amplitude estimated marginal means evaluated at age = 11.172, IQ = 110.68 for the ASD (blue) and NT (green) groups depicting the **A** group x motion-type interaction in the P1 period, **B** group x motion-type x task interaction in the N1 period and **C** group x motion-type x task interaction in the P2 period. Error bars = 95% CI

For the P1 component (136-156 ms), there were no significant main effects of group (NT, ASD), hemisphere (left-PO7, right-PO8), task (unattended, attended) or motion type (UM, IM, SM) after controlling for age and IQ. There was also no main effect of IQ or IQ-related interactions. There was an early emerging group x motion-type interaction (F(2, 98) = 3.108, *p* = 0.049, η_p_^2^ = 0.060). NT participants had an early suppression of the P1 response to biological motion stimuli (UM and IM) relative to SM, while individuals with ASD showed the reverse pattern (Fig. 3a). There was also a main effect of age (F(1,49) = 14.874, *p* < 0.001, η_p_^2^ = 0.233) with younger participants showing higher amplitude P1 (Additional file 4). No other effects were significant.

For the N1 component (207–227 ms), there were no main effects of group, task, motion type, age or IQ. There was a main effect of hemisphere (F(1,49) = 4.061, *p* = 0.049, η_p_^2^ = 0.077), with slightly increased amplitudes to all motion types in the right relative to left hemisphere. There was a group x motion-type x task interaction (F(1.621,79.409) = 4.780, *p* = 0.016, η_p_^2^.089,*Greenhouse–Geisser-corrected for violation of Mauchly’s test of sphericity). In the unattended condition, NT participants had greater magnitude N1 to the UM with stepwise reduction in response to the IM and finally SM, whereas ASD participants showed an opposite pattern with reduced magnitude responses to UM and elevated responses to IM relative to SM (Fig. 3b). In the attended condition, both NT and ASD participants showed equivalent responses to UM and SM. However, NT participants showed relative suppression in response to IM whereas ASD participants had an augmented N1 to IM (Fig. 3b). There was also a motion-type x age interaction (F(2,98) = 4.099, *p* = 0.020, η_p_^2^ = 0.077) whereby biological motion-specific processing effects tended to diminish with increasing age (Additional file 4). No other interactions were significant.

For the P2 component (370–450 ms), there were no main effects of group, task, motion type, age or IQ. There was a group x motion type x task interaction (F(2,98) = 6.818, *p* = 0.002, η_p_^2^ = 0.122). In the unattended condition, the NT group showed continuation of the pattern observed in the N1 period extending into P2 suppression, such that the lowest magnitude P2 was observed to the UM, followed sequentially by IM and SM. ASD participants had equivalent P2 to UM and SM with atypically elevated response to IM (Fig. 3c). In the attended condition, NT participants showed the most P2 suppression to SM, with more equivalent responses to UM and IM. In contrast, the ASD group continued to have atypical suppression of the P2 to IM relative to the other two motion types. ASD participants also showed greater magnitude P2 to all motion types in the attended condition relative to NT controls (Fig. 3c). There was also a hemisphere x motion-type x age interaction (F(1.646,80.668) = 5.619, *p* = 0.008, η_p_^2^ = 0.103, *Greenhouse–Geisser-corrected for violation of Mauchly’s test of sphericity). Older participants showed more rightward lateralization and less differentiation across motion types than younger participants. Younger participants showed more leftward lateralization and a stepwise decrement in P2 with the highest magnitude P2 to SM, followed in order by IM and UM (Additional file 4). No other main effects or interactions were significant in this time window.

#### Stage two analysis: whole brain exploratory statistical cluster plots

**Fig. 4:**
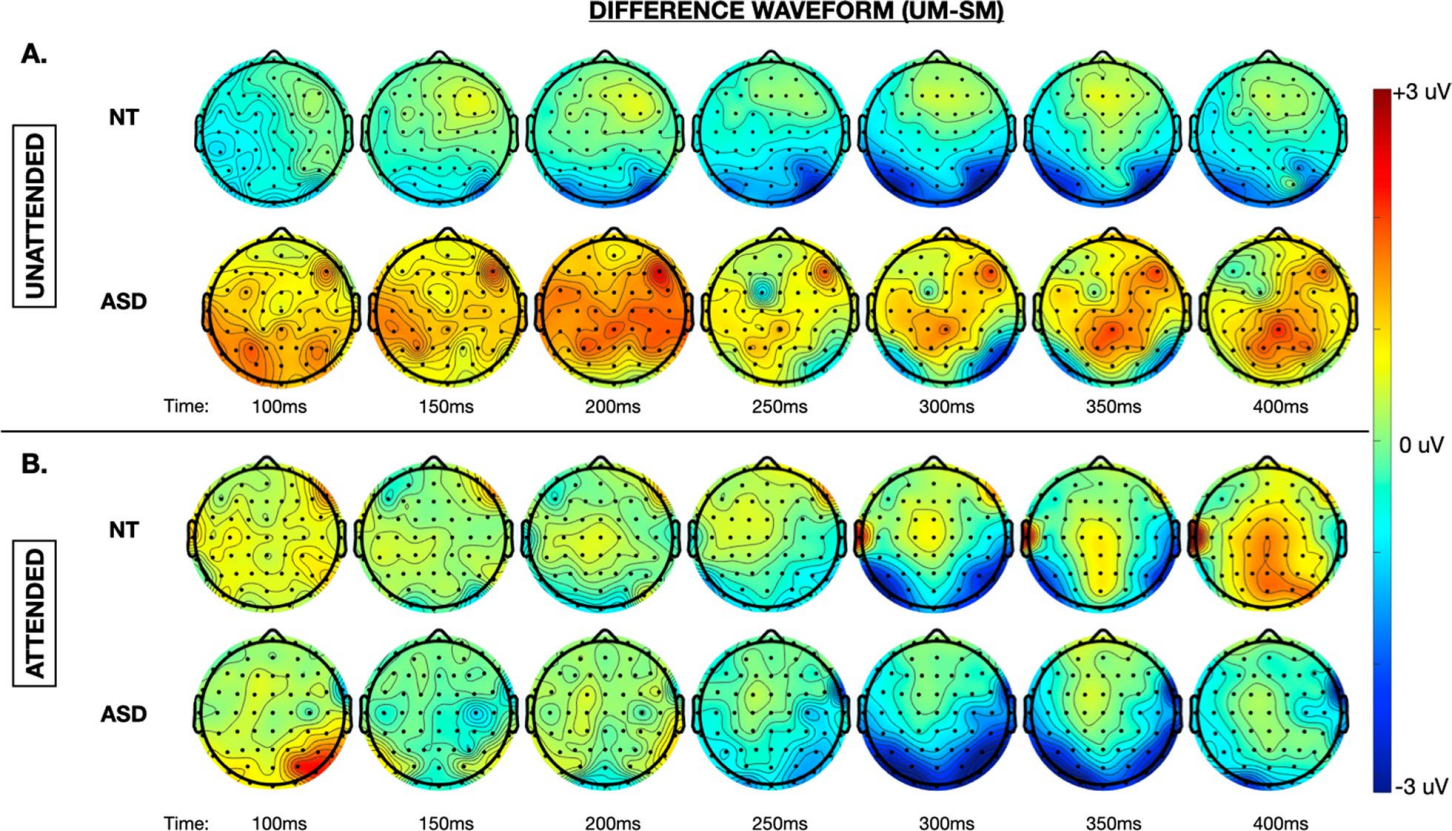
Topographic representation of the difference in instantaneous amplitude of evoked response between UM and SM in the **A** unattended and **B** attended for NT and ASD participants at 50-ms intervals between 100 and 400 ms post-stimulus onset

**Fig. 5:**
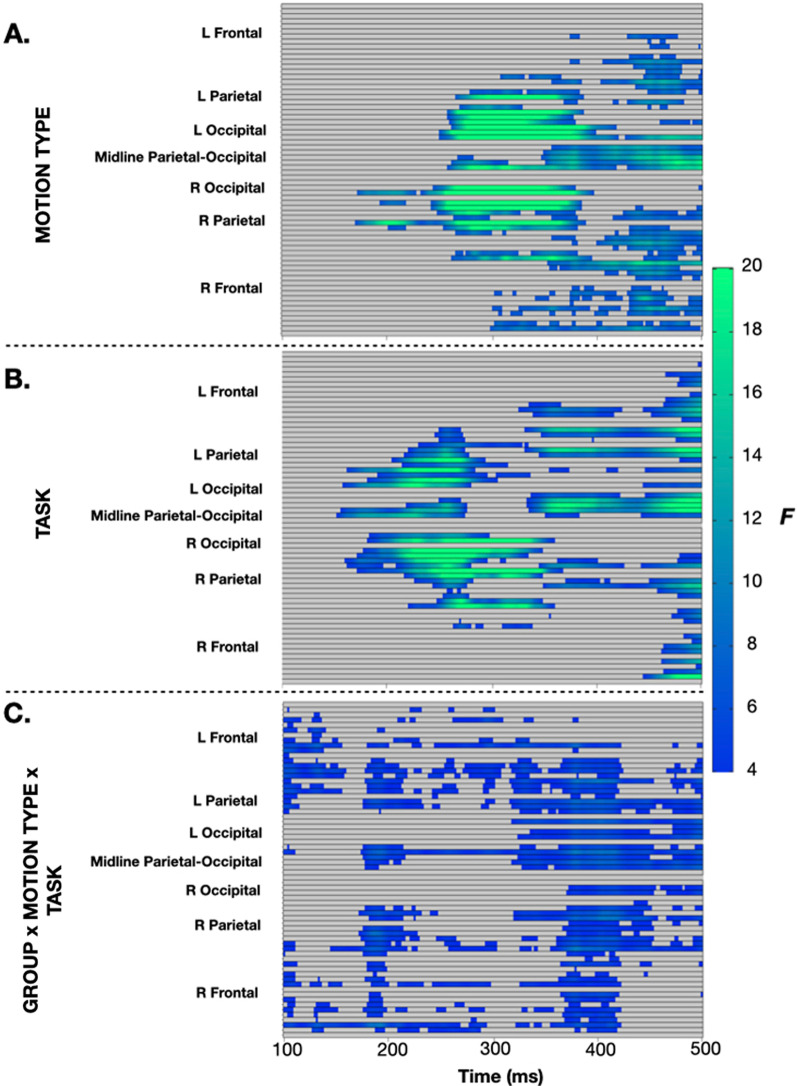
Results of the stage two exploratory cluster-corrected factorial mass analysis across all electrodes and time points (100–500 ms) with a between-subjects factor of group (NT, ASD) and within-subject factors of motion type (UM, SM, IM) and task (unattended, attended). **A** Regions and time points showing a main effect of motion type. **B** Regions and time points showing a main effect of task. **C** Regions and time points showing a group x motion-type x task interaction. No other main effects or interactions were significant

Dynamics of biological motion processing across the whole scalp are depicted by topographic maps of difference in evoked response to UM as compared to SM for each group and attentional task (Fig. 4). The ASD group displayed notably more attention-related modulation of whole brain activity than the NT participants, with selective response to UM over SM corresponding much more with NT activation patterns only in the attended condition (Fig. 4). (Full topographic maps for both groups across all conditions are depicted in Additional file 5.) To explore this, we implemented an exploratory cluster-corrected factorial mass analysis across all electrodes and time points (100–500 ms) with a between-subjects factor of group (NT, ASD) and within-subject factors of motion type (UM, SM, IM) and task (unattended, attended) (Fig. 5). This revealed a main effect of motion type whereby greater negativity was generated in the left, midline, and right parietal–occipital regions by UM stimuli, most pronounced between 250 and 350 ms post-stimulus onset (Fig. 5a). Task-related effects were noted in similar regions originating slightly earlier between 200 and 300 ms with attention to biological motion generating a greater difference in amplitude of evoked response to UM as compared to SM (Fig. 5b). Smaller magnitude group x motion-type x task interaction effects were noted in bilateral frontal regions between 100 and 150 ms, followed by bilateral frontal–parietal and midline occipital–parietal regions between 200 and 250 ms, and finally right frontal along with bilateral parietal and midline parietal and occipital regions between 375 and 450 ms (Fig. 5c).

#### Supplemental analysis: sex-related effects in NT development

Given the skewed sex ratio in the ASD group, we explored any effect of sex by conducting a post hoc analysis separately on the NT group only. Data from NT participants were analyzed using a generalized linear mixed model with a between-groups factor of sex and within-subject factors of motion type (UM, SM, IM), task (unattended, attended), and hemisphere (right, left), as well as age and IQ as continuous covariates, across the same three P1, N1, and P2 time windows used in the primary analysis. There was a mildly significant hemisphere x task x sex interaction in the P2 period F(1,27) = 4.971, *p* = 0.034, η_p_^2^ = 0.155), with males showing more attention-related modulation in the right hemisphere. However, there were no other sex-related interactions nor main effect of sex in any of the time windows tested and notably no effects involving motion type, indicating that sex was likely not a contributor to the ASD-related effects in biological motion processing observed in the primary analysis (Additional file 6).

### EEG–phenotype correlations

**Fig. 6:**
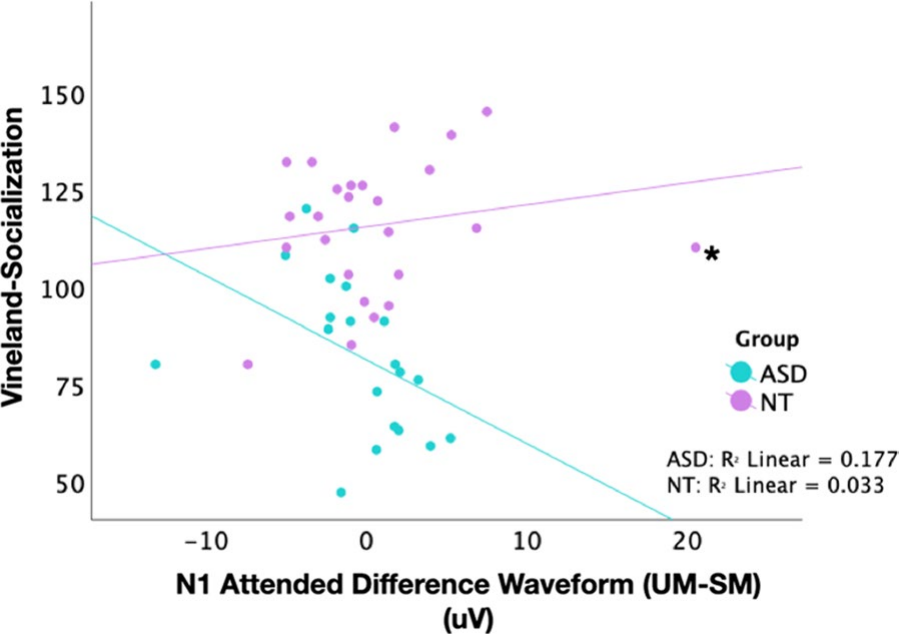
Scatter plots depicting the correlations between Vineland Socialization Standard Scores and difference in mean amplitude for the UM versus SM waveforms in the N1 period for ASD (turquoise) and NT (purple) participants. *Denotes an NT outlier that was explored. Data was ultimately included in the analysis

Multiple linear regression analyses of the relationships between the difference in mean amplitude for the UM vs. SM waveforms in each of the P1, N1, and P2 periods and tasks (unattended, attended) with Vineland Adaptive Behavior Scale Socialization domain scores was conducted separately within the ASD and NT groups. Figure 6 depicts significant relationships. The results of the full analysis are also included in Additional file 7. Within the ASD group, greater magnitude difference in the N1 evoked by UM as compared to SM stimuli in the attended condition predicted better functional social communication (*β* = − 4.745, *t* = − 2.684, *p* = 0.019). In the NT group, no relationships between any of the measures tested and socialization were observed. Although not part of the a priori design, in response to a reviewer suggestion, we also explored correlations between the same electrophysiologic measures and other Vineland subscales (General Adaptive Composite, Communication, Daily Living). The results are presented in Additional file 8. Within the ASD group, the difference in N1 mean amplitude evoked by UM vs SM during the attended task also correlated with each of the other subscales; however, this is perhaps unsurprising given that these subscales themselves are highly intercorrelated.

## Discussion

In this study, we sought to address gaps in the understanding of biological motion processing in ASD by delineating the spatiotemporal dynamics of biological motion processing and their modulation by attention among children with and without ASD. Despite behavioral similarity to NT children and adolescents in their ability to discriminate biological from non-biological motion, individuals with ASD demonstrated distinct differences in their patterns of unintentional and intentional neural processing of biological motion stimuli. These findings suggest atypical development of low-level biological motion processing mechanisms with potential implications for higher-order social-cognitive functions in ASD.

### Development of biological motion processing

A previous study assessed biological motion processing in neurotypical adults and revealed three waves of activity over bilateral parieto-occipital sites; the earliest phase of activity (100–200 ms) was unaffected by task, the second phase (200–350 ms) was amplified during the attended task, and the third phase (400 + ms) was only evident in the attended task condition [58]. Children and adolescents in the present study demonstrated topographic similarity to the biological motion processing observed in the previous adult study, albeit with evidence of continued developmental tuning. With increasing age, we observed progressively decreased P1 amplitude and decreased differentiation in processing of biological and non-biological motion during the N1 and P2 periods. This appeared similar to the age-related linear decrements in occipito-temporal P1-N1 intensity described in a previous study of childhood biological motion development by Hirai and colleagues [74]. We also observed maturational effects on hemispheric lateralization of biological motion processing, with more pronounced rightward lateralization of biological motion-specific processing during the P2 component in the older as compared to younger participants. Consistent with this, a common finding in the adult literature is an amplified response to biological motion stimuli in the right hemisphere relative to the left, although evidence of biological motion processing is observed bilaterally as well [58, 75–77]. While previous behavioral studies have shown that aspects of biological motion processing appear to be innate and present from birth [5], our findings of age-related effects on biological motion processing do suggest that the visual system continues to tune its processing of these fundamental stimuli during development. Given the dynamic nature of the human brain in general, it is perhaps not surprising that biological motion processes continue to mature through childhood and adolescence in typical development.

Of note, no relationship between IQ and any of the factors tested was observed, echoing the conclusions of previous meta-analyses that cognitive factors do not appear to substantially impact development of biological motion processing [25, 26]. Likewise, no sex-related effects were observed in the processing of biological vs. non-biological motion. Although sex differences have been occasionally described in biological motion processing among adults such that females may have more activation to biological motion over scrambled motion in social regions (i.e., amygdala, medial temporal gyrus, temporal pole), these differences are notably less pronounced in children [78], which may coincide with the absence of sex-related effects among NT children observed here.

### Biological motion processing in autism spectrum disorder

When processing biological motion stimuli, both the NT and ASD groups were able to discriminate upright and inverted biological motion from scrambled motion with roughly equivalent accuracy. The ASD group showed slightly poorer behavioral performance as compared to NT children on both the attended and unattended tasks, although this difference did not reach a threshold of statistical significance and was not modulated to a significant extent by any of the experimental conditions under study. Thus, on a behavioral level, biological motion processing appears fairly intact in ASD. However, previous studies have reported a dissociation of neural deficits from performance deficits, suggesting differential deployment of neural circuitry in biological motion processing in ASD [19, 30]. Consistent with this, distinctive differences in neurophysiologic mechanisms of biological motion processing between NT and ASD participants in our study were apparent beginning in the P1 period and were accentuated in the N1 extending through P2 periods, particularly when subjects were not actively attending to biological motion. In general, NT participants showed automatic biological motion-related processing with N1 augmentation and P2 suppression that was most pronounced to upright motion followed sequentially by IM and SM. In contrast, ASD participants did not show the same automatic biological motion-related N1 augmentation and P2 suppression pattern. Hirai and colleagues have also reported similar unattended biological motion processing dynamics in children; in NT controls but not ASD participants, they found augmentation of right hemisphere amplitudes in response to a point-light display of a walking person relative to its scrambled counterpart, appearing to emerge between 100 and 200 ms post-stimulus onset and becoming most pronounced between 250 and 500 ms [79]. Our findings of motion-type effects that emerge in the NT group around 140 ms in the P1 component and become more evident in the 200–450 ms N2 and P2 components are therefore highly consistent with their findings.

Notably, differences between the NT and ASD groups during processing of UM and SM were much more subtle when participants were explicitly directed to discriminate biological from non-biological motion. Even when NT participants were focused on a distractor task and not explicitly cued to the presence of biological motion, they had amplified neural responses distinguishing UM and SM, likely suggesting an automatic and involuntary allocation of attentional resources to biological motion processing. In contrast, the ASD group demonstrated this pattern of substantial biological motion-related segregation in visual evoked responses predominantly only during the explicitly attended task, perhaps paralleling other studies showing that children and adolescents with ASD are not as susceptible to the automatic capture of attention by socially salient stimuli [51, 80]. Thus, these findings lend support for the model that individuals with ASD may not have the same degree of tuning to biological motion processing at baseline but are able to process biological motion when actively attending. This electrophysiologic evidence, along with previous studies demonstrating correlation between differential fixations and biological motion task performance, highlights the critical need to consider attentional factors during biological motion paradigms in studies of ASD.

Similar spatiotemporal dynamics of biological motion processing have been localized to the STS during the same tasks in NT adults [58]; however, given that developmental changes in cortical sources of some ERP components have been described, we cannot specifically evaluate the role of STS-related dysfunction in biological motion processing based solely on the sensor-level data from children in this study. Despite this obvious limitation, our current findings of ASD-related dysfunction in these biological motion-related processes certainly do not contradict and in fact may coincide with the neuroimaging literature that has implicated hypoactivation of occipito-temporal regions such as pSTS in disordered biological motion processing in ASD [19, 31, 50, 81]. For example, a recent functional neuroimaging study by Alaerts and colleagues using similar biological motion stimuli and attentional manipulations reported STS hypoactivation to biological motion over scrambled motion in the ASD group relative to NT controls in the unattended condition, although both groups did show some evidence of biological motion processing [50]. However, in contrast to our finding that explicitly attending to biological motion stimuli partially ameliorates deficits in automatic biological motion processing mechanisms among those with ASD, Alaerts and colleagues revealed instead a lack of enhanced STS neural activity for the explicit attention condition as compared to the passive condition in ASD adults [50]. Although both studies potentially implicate similar brain regions in biological motion processing deficits among those with ASD and emphasize the importance of attention as a modulating factor, the attentional influence appears to be reversed. It is unclear whether this discrepancy in study findings is attributable to age or to differences inherent in functional imaging as compared to electrophysiologic modalities of measurement, especially considering that cortical source localization was not performed in this study so any estimation of cortical generators represents an extrapolation from previous adult-only data. This potentially could be resolved by future studies employing both techniques in the same cohort.

Interestingly, we also noted atypical prominence across multiple components of the visual evoked potential to IM stimuli irrespective of task in the ASD group. Although inversion of biological motion point-light display stimuli affects both perception of biological motion and preferential attendance, IM stimuli do retain overall global configuration of biological motion point-light displays and are therefore more perceptually salient than SM, but not UM stimuli. The preservation of some biological motion information [5, 6, 64, 82] likely explains why the average visual evoked response amplitude for IM was intermediate between UM and SM stimuli in NT participants. In accordance with this, similar segregations in functional activity between UM, IM and SM have also been reported within the posterior STS [64]. Studies of inverted biological motion stimuli in ASD, though, have been fairly limited and present some discrepant findings. One study by Klin and colleagues found that toddlers with ASD did not exhibit the same passive viewing preference for upright over inverted biological motion point-light displays that both NT children and children with non-ASD developmental delay exhibited [51]. Another more recent study of children and adolescents age 2–18 did not reveal significant differences in preference for upright over inverted motion between ASD and NT groups, but it was acknowledged that synchronous presentation of sound with the motion stimuli may have contributed to this null finding [83]. As reflexive orientation of attention to UM over IM stimuli has been shown to mature over early development [84], the atypical processing of IM within our ASD sample may represent either delay or deviation from typical development in children with ASD. However, given unresolved controversy surrounding the potential for atypical processing of inverted stimuli in this population, further investigation is warranted to better understand the developmental mechanisms of inverted biological motion processing and their implications for individuals with ASD.

Finally, the emergence of biological motion processing group differences in the very early sensory-perceptual period suggests that biological motion processing differences in ASD are not limited to higher-order social-cognitive dysfunctions such as identification of intentions or affective states and lends support for the possibility that specific biological motion and/or other low-level sensory deficits early in development may feed the social-cognitive dysfunctions that are a hallmark of the disorder. Indeed, the idea of shared circuitry underlying neural dysfunction in both biological motion processing and social cognition has been proposed previously in a growing body of literature [9, 22–24, 35, 36], and several studies have reported associations of weaker biological motion processing with higher autism symptom severity [35, 36, 81]. Here, we found that greater biological motion-specific processing in the N1 period for the ASD group was associated with better socialization adaptive ability. It is difficult to speculate on why socialization scores in those with ASD only would be predicted by explicitly attended biological motion processing in the N1 period, and the cross-sectional design of the present study also precludes the ability to draw conclusions on directional causal relationships. However, these findings convincingly raise the possibility that discrepant development of low-level visual processing mechanisms may contribute to social atypicality in ASD. Given the potential relevance of basic biological motion processing mechanisms to higher-order aspects of social communication and cognition such as imitation, gesture production and empathy, these relationships warrant continued attention in future investigations.

## Limitations

The current analysis was focused on the early sensory-perceptual period; however, there are substantial effects related to biological vs. non-biological motion that appear to persist beyond the end of this period, and specific investigation of post-perceptual processing may also be of interest. Additionally, generators identified in the previous adult study have implied a role for STS in the processing of biological motion [58], but it would be useful to directly assess electrophysiologic and functional imaging correlates of biological motion processing within the same subjects to clarify this relationship, especially given that age-related changes in generator configurations have been noted, particularly in the auditory modality [85–87]. Extensive studies from our group and others exploring visual perception in detail across a wide developmental period reveal large changes in the amplitude of visual evoked potential components with age such that amplitudes tend to decrease markedly over the course of childhood into adolescence; however, the general morphology and topography do not appear to change to the same extent [85, 88]. Nonetheless, it is entirely plausible that more subtle developmental changes across the broad age range included in this study would be missed by defining electrode sites of interest based on previous adult findings, and the study is not powered sufficiently within narrower age bands to comprehensively explore these possibilities. With regard to the study sample, due to task demands participation was limited to high-functioning individuals with ASD and the findings may not be generalizable to children with intellectual disability. Lastly, while this study helps to clarify the role of explicit attention in the processing of biological motion in children and adolescents with ASD, there is a high comorbidity in ASD and ADHD diagnoses [89] and children with ADHD have been shown to have similar biological motion-related N2 effects [90]. The NT group did not include children with ADHD consistent with a conservative definition of NT development, and although children with ASD and comorbid ADHD were not explicitly excluded, only one participant in the ASD group endorsed formal ADHD diagnosis. As such, by a priori design the sample was not adequately powered to definitively separate the relative contribution of these two developmental diagnoses. Though outside the scope of this study, exploration of biological motion processing in populations with and without ADHD would be of substantial interest for future studies. These findings, along with a growing body of literature implicating covert and overt attentional mechanisms in multiple aspects of sensory processing [91], emphasize the need to consider a possible role of disrupted attentional mechanisms when designing and executing paradigms to investigate sensory perception in ASD along with other developmental disabilities. Attentional factors certainly cannot be the only source of inconsistency in findings across studies nor of interindividual variability in biological motion processing observed in this and other studies. However, it does suggest that attentional factors are critical to consider alongside the other phenotypic dimensions on which children with autism spectrum differ.

## Conclusions

Overall, this study addresses a critical gap by directly assessing the impact of attention on neural mechanisms of low-level biological motion discrimination in NT development and in children with ASD. Although children with ASD were able behaviorally to discriminate biological motion with roughly similar accuracy to NT children, electrophysiologic investigation demonstrates reduced automatic selective processing of upright biological vs. scrambled motion stimuli that improves with explicit attention to biologic motion. The association between neural mechanisms of biological motion processing and a measure of real-world social adaptive function in children lends credence to a model whereby biological motion processing in the early sensory-perceptual period may contribute to aspects of atypical higher-order social cognition among children and adolescents with ASD.

## Supplementary Information

**Additional file 1**: Interpolated channels and accepted trials.

**Additional file 2**: d-prime values by group and condition**.**

**Additional file 3**: Scatter plot depicting in d’ in the unattended (blue) and attended (orange) tasks with age (top panel) and IQ (bottom panel) for A) NT and B) ASD participants.

**Additional file 4**: Grand average visual evoked potentials at bilateral parieto-occipital sites (PO7 and PO8) obtained in A) younger participants (age 6-10) and B) older participants (age 11-16) to UM (orange), SM (purple), and IM (blue) collapsed across groups and tasks. C) Difference waveform of the evoked potential to UM minus SM in younger (dotted line) and older (solid line) participants.

**Additional file 5**: Topographic representation of the instantaneous amplitude of evoked response to each of SM, UM and IM in the unattended and attended tasks for NT and ASD participants at 50-ms intervals between 100 and 400ms post-stimulus onset.

**Additional file 6**: Supplemental analysis: Sex-related effects in NT development.

**Additional file 7**: EEG–phenotype correlations-Vineland Socialization Subscales.

**Additional file 8**: EEG–phenotype correlations (additional Vineland subscales).

## Data Availability

The datasets used and/or analyzed during the current study are available from the corresponding author on reasonable request.

## Abbreviations

ASD: Autism spectrum disorder
EEG: Electroencephalographic
NT: Neurotypical
STS: Superior temporal sulcus
UM: Upright motion
SM: Scrambled motion
IM: Inverted motion
ADHD: Attention deficit hyperactivity disorder
