# Supplementary material for: Attentional influences on neural processing of biological motion in typically developing children and those on the autism spectrum
Source: Mol Autism. 2022 Jul 18;13:33. doi: 10.1186/s13229-022-00512-7 (PMC9290301; doi:10.1186/s13229-022-00512-7)
Supplement: Supplementary file 1 — Additional file 1: Interpolated channels and accepted trials. [file 13229_2022_512_MOESM1_ESM.docx]

**Additional File 1. Interpolated channels and Accepted trials**

|  |  |  | **NT (n=35)** | **ASD (n=27)** | **Significance** |
| --- | --- | --- | --- | --- | --- |
| **Interpolated Channels** | Unattended |  | 5.14(2.6) | 4.89(2.8) | p=.712 |
|  | Attended |  | 4.51(2.9) | 4.89(1.87) | p=.542^a^ |
| **Accepted Trials (%)** | Unattended | SM | 91.7(12.6) | 93.1(10.7) | p=.651 |
|  |  | UM | 91.2(13.8) | 92.6(13.6) | p=.681 |
|  |  | IM | 91.6(13.6) | 93.5(10.5) | p=.553 |
|  | Attended | SM | 90.9(9.4) | 90.2(11.4) | p=.794 |
|  |  | UM | 90.2(10.6) | 90.4(10.8) | p=.961 |
|  |  | IM | 90.4(10.8) | 91.2(10.5) | p=.770 |

^a^Levene’s test significant, equal variances not assumed
