# Supplementary material for: Attentional influences on neural processing of biological motion in typically developing children and those on the autism spectrum
Source: Mol Autism. 2022 Jul 18;13:33. doi: 10.1186/s13229-022-00512-7 (PMC9290301; doi:10.1186/s13229-022-00512-7)
Supplement: Supplementary file 2 — Additional file 2: d-prime values by group and condition. [file 13229_2022_512_MOESM2_ESM.docx]

**Additional File 2. d-prime values by group and condition**

| **Task** | **d’**  **mean (SD)** | | | | | |
| --- | --- | --- | --- | --- | --- | --- |
|  | **NT** | **ASD** | **NT** | | **ASD** | |
| **Attended** | 1.50(.78) | 1.07(.79) | **UM**  1.66(.76) | **IM**  1.19(.72) | **UM**  1.18(.83) | **IM**  .89(.69) |
| **Unattended** | 1.50(.78) | 1.31(.81) |  |  |  |  |
|  |  |  |  |  |  |  |
