# Supplementary material for: Attentional influences on neural processing of biological motion in typically developing children and those on the autism spectrum
Source: Mol Autism. 2022 Jul 18;13:33. doi: 10.1186/s13229-022-00512-7 (PMC9290301; doi:10.1186/s13229-022-00512-7)
Supplement: Supplementary file 3 — Additional file 3: Scatter plot depicting in d’ in the unattended (blue) and attended (orange) tasks with age (top panel) and IQ (bottom panel) for A) NT and B) ASD participants. [file 13229_2022_512_MOESM3_ESM.docx]

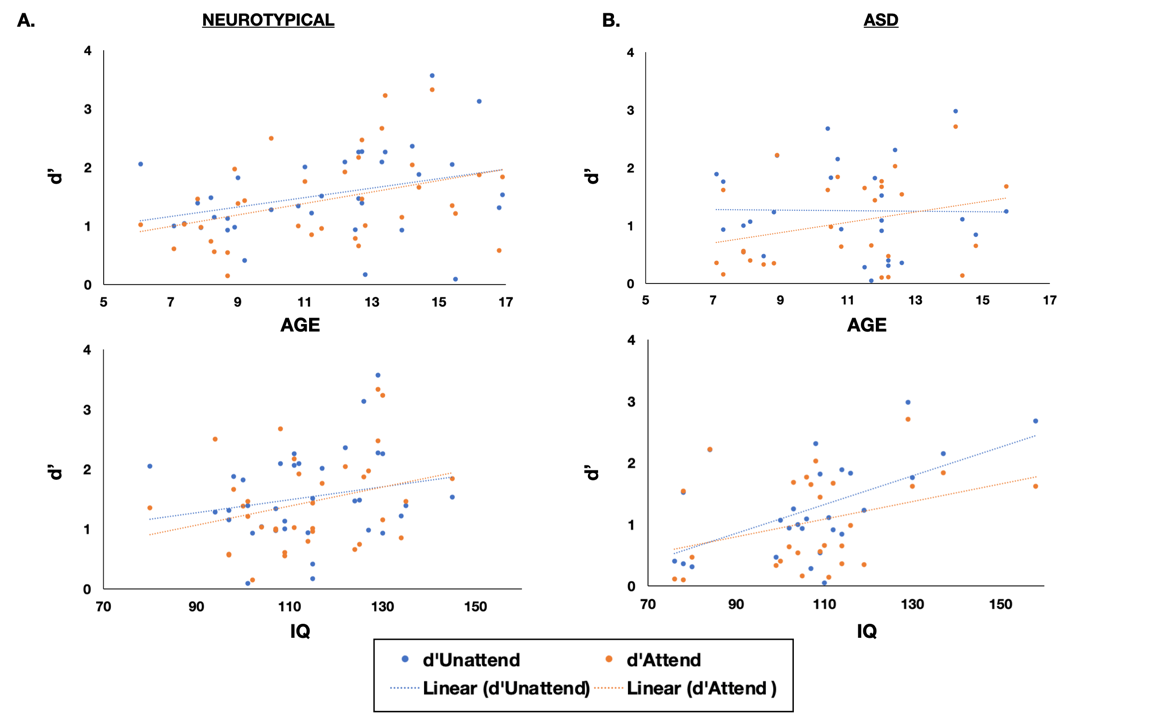


**Additional File 3.** Scatter plot depicting in d’ in the unattended (blue) and attended (orange) tasks with age (top panel) and IQ (bottom panel) for **A)** NT and **B)** ASD participants.
