# Supplementary material for: Attentional influences on neural processing of biological motion in typically developing children and those on the autism spectrum
Source: Mol Autism. 2022 Jul 18;13:33. doi: 10.1186/s13229-022-00512-7 (PMC9290301; doi:10.1186/s13229-022-00512-7)
Supplement: Supplementary file 4 — Additional file 4: Grand average visual evoked potentials at bilateral parieto-occipital sites (PO7 and PO8) obtained in A) younger participants (age 6-10) and B) older participants (age 11-16) to UM (orange), SM (purple), and IM (blue) collapsed across groups and tasks. C) Difference waveform of the evoked potential to UM minus SM in younger (dotted line) and older (solid line) participants. [file 13229_2022_512_MOESM4_ESM.docx]

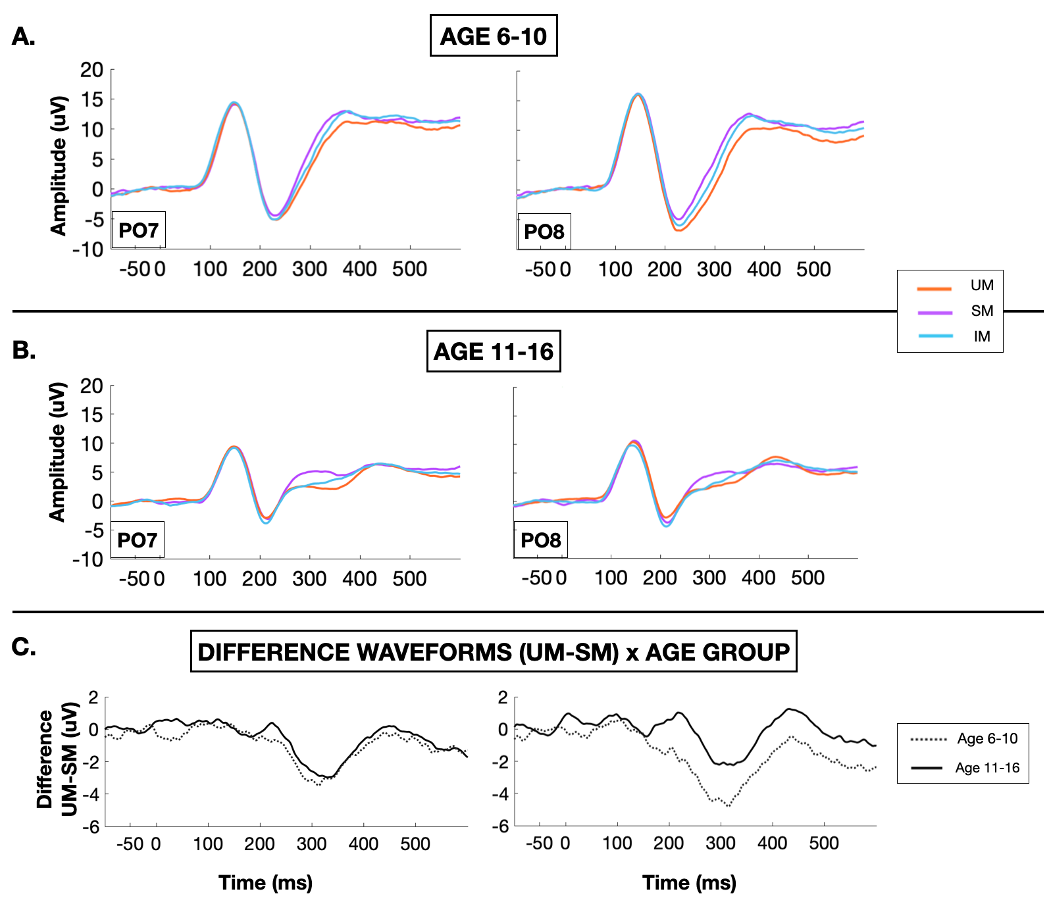


**Additional File 4.** Grand average visual evoked potentials at bilateral parieto-occipital sites (PO7 and PO8) obtained in **A)** younger participants (age 6-10) and **B)** older participants (age 11-16) to UM (orange), SM (purple), and IM (blue) collapsed across groups and tasks. **C)** Difference waveform of the evoked potential to UM minus SM in younger (dotted line) and older (solid line) participants.
