# Supplementary material for: Attentional influences on neural processing of biological motion in typically developing children and those on the autism spectrum
Source: Mol Autism. 2022 Jul 18;13:33. doi: 10.1186/s13229-022-00512-7 (PMC9290301; doi:10.1186/s13229-022-00512-7)
Supplement: Supplementary file 5 — Additional file 5: Topographic representation of the instantaneous amplitude of evoked response to each of SM, UM and IM in the unattended and attended tasks for NT and ASD participants at 50-ms intervals between 100 and 400ms post-stimulus onset. [file 13229_2022_512_MOESM5_ESM.docx]

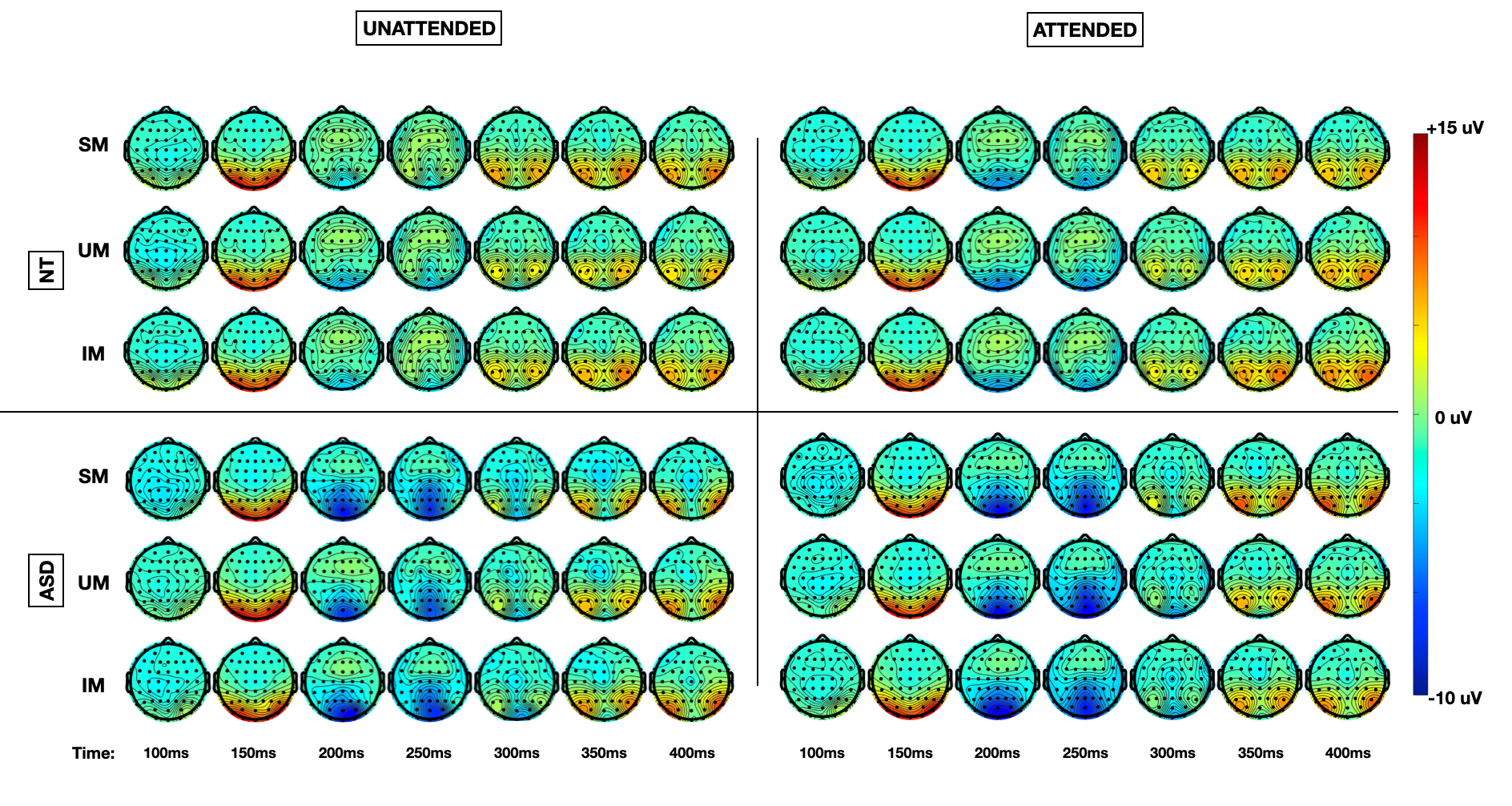


**Additional File 5.** Topographic representation of the instantaneous amplitude of evoked response to each of SM, UM and IM in the unattended and attended tasks for NT and ASD participants at 50ms intervals between 100-400ms post-stimulus onset.
