# Supplementary material for: Attentional influences on neural processing of biological motion in typically developing children and those on the autism spectrum
Source: Mol Autism. 2022 Jul 18;13:33. doi: 10.1186/s13229-022-00512-7 (PMC9290301; doi:10.1186/s13229-022-00512-7)
Supplement: Supplementary file 6 — Additional file 6: Supplemental analysis: Sex-related effects in NT development. [file 13229_2022_512_MOESM6_ESM.docx]

## Additional File 6. Supplemental analysis: Sex-related effects in NT development

|  | | **P1**  **(136-156ms)** | **N1**  **(207-227ms)** | **P2**  **(370-450ms)** |
| --- | --- | --- | --- | --- |
|  | | **F (p value)** | | |
| **Main Effects** | | | | |
| Sex | df (1,27) | 1.756 (p=.196) | 1.193 (p=.284) | 2.308 (p=.079) |
| **Within Subject x Between Subject Interactions** | | | | |
| Hemisphere x Sex | df (1,27) | .538 (p=.470) | 3.563 (p=.070) | 2.392 (p=.134) |
| Task x Sex | df (1,27) | .662 (p=.423) | .164 (p=.689) | .807 (p=.377) |
| Motion-type x Sex | df (2,54) | 2.637 (p=.081) | .459 (p=.634) | .380 (p=.685) |
| Hemisphere x Task x Sex | df (1,27) | .030 (p=.863) | .3.679 (p=.066) | **4.971 (p=.034)*** |
| Hemisphere x Motion-type x Sex | df (2,54) | .161 (p=.776^a^) | .268 (p=.663^a^) | .131 (p=.762^a^) |
| Task x Motion-type x Sex | df (2,54) | .698 (p=.466^a^) | .920 (p=.390^a^) | 1.082 (p=.343) |
| Hemisphere x Task x Motion-type x Sex | df (2,54) | 2.540 (p=.108^a^) | .271 (p=.646^a^) | .096 (p=.804^a^) |

Note: Degrees of freedom (df) reported as uncorrected (sphericity assumed) values though where appropriate p values marked as reflecting ^a^Greenhouse-Geisser correction for violation of Mauchly’s test of sphericity ^a^Greenhouse-Geisser corrected for violation of Mauchly’s test of sphericity; * p<.05; n_female_=16,n_male_=15
