# Supplementary figures and images for: Attentional influences on neural processing of biological motion in typically developing children and those on the autism spectrum
Source: Mol Autism. 2022 Jul 18;13:33. doi: 10.1186/s13229-022-00512-7 (PMC9290301; doi:10.1186/s13229-022-00512-7)

**Additional File 7. EEG-Phenotype Correlations-Vineland Socialization Subscales**

**
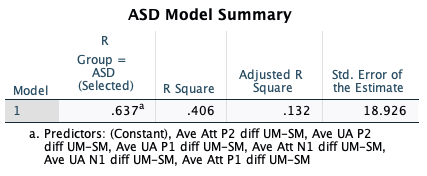
**

**
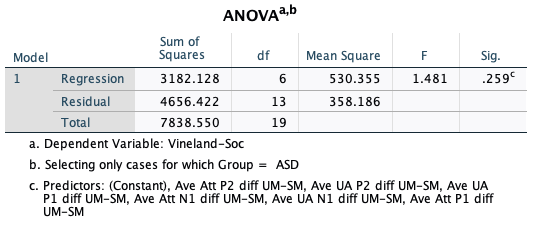
**

**
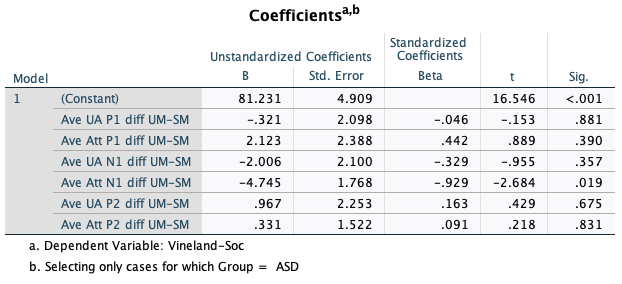
**

**
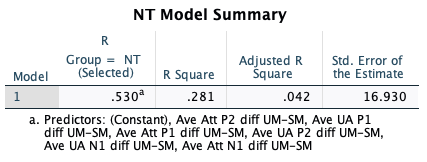
**

**
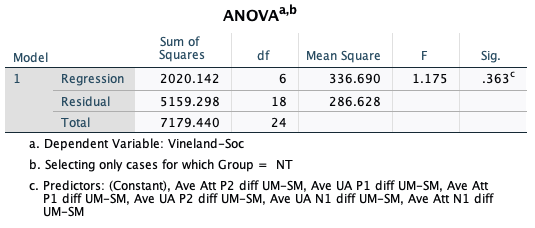
**

**
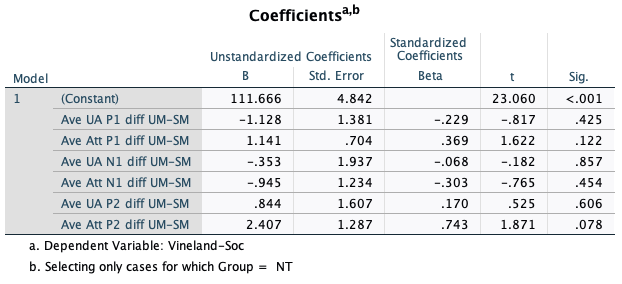
**

Supplement: Supplementary file 7 — Additional file 7: EEG–phenotype correlations-Vineland Socialization Subscales. [file 13229_2022_512_MOESM7_ESM.docx]

**Additional File 8. EEG-Phenotype Correlations (additional vineland subscales)**

**
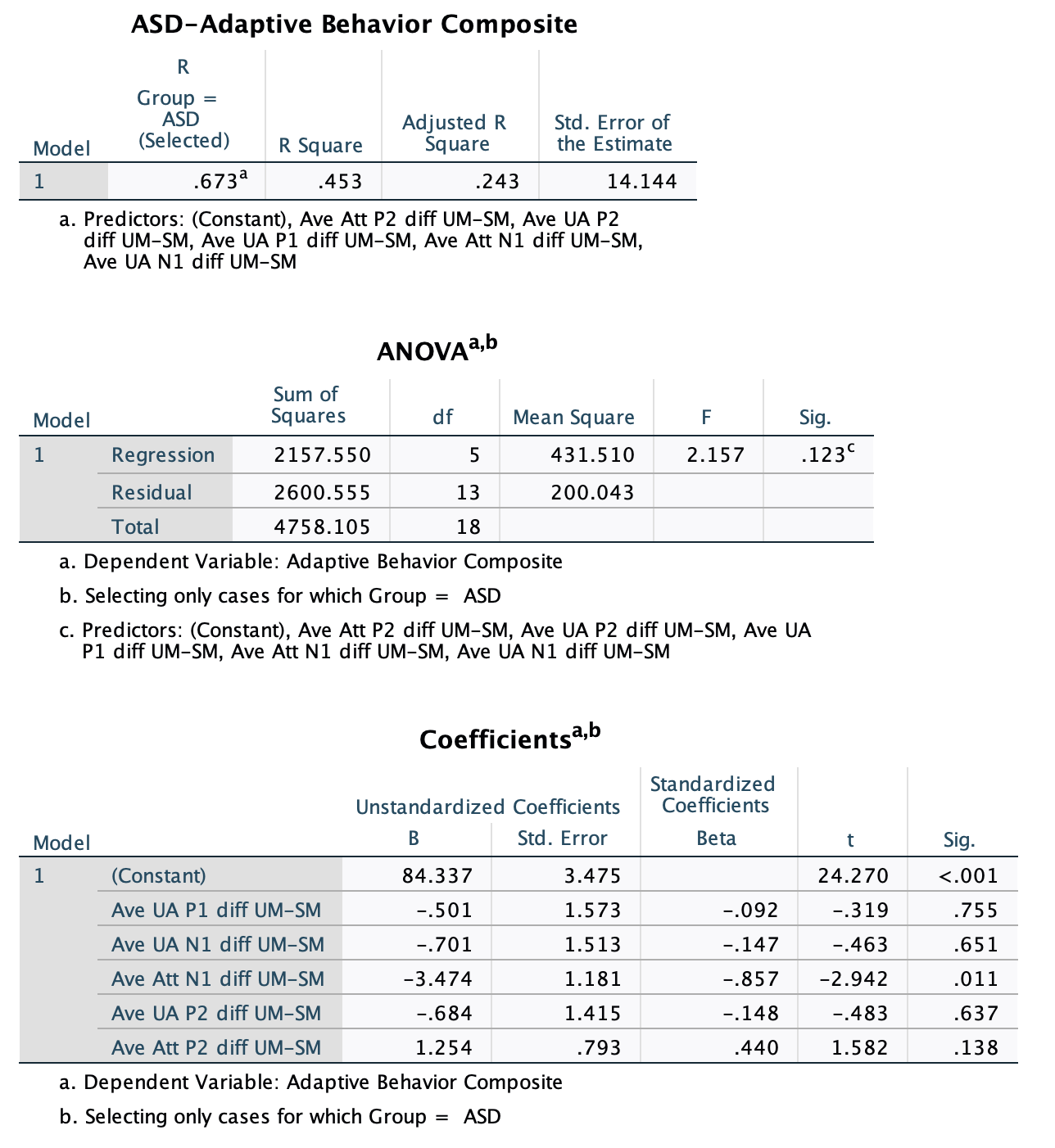
**


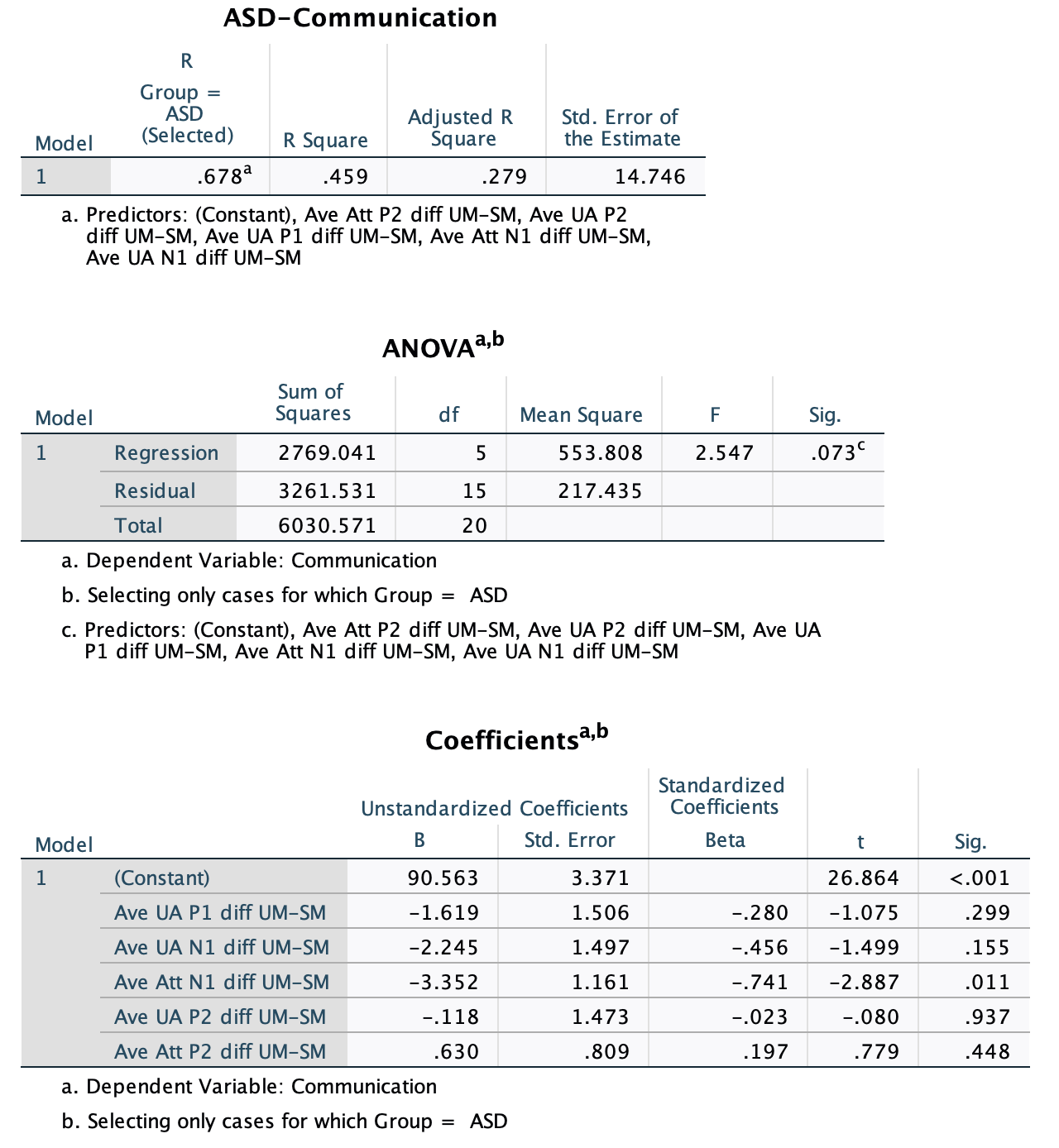


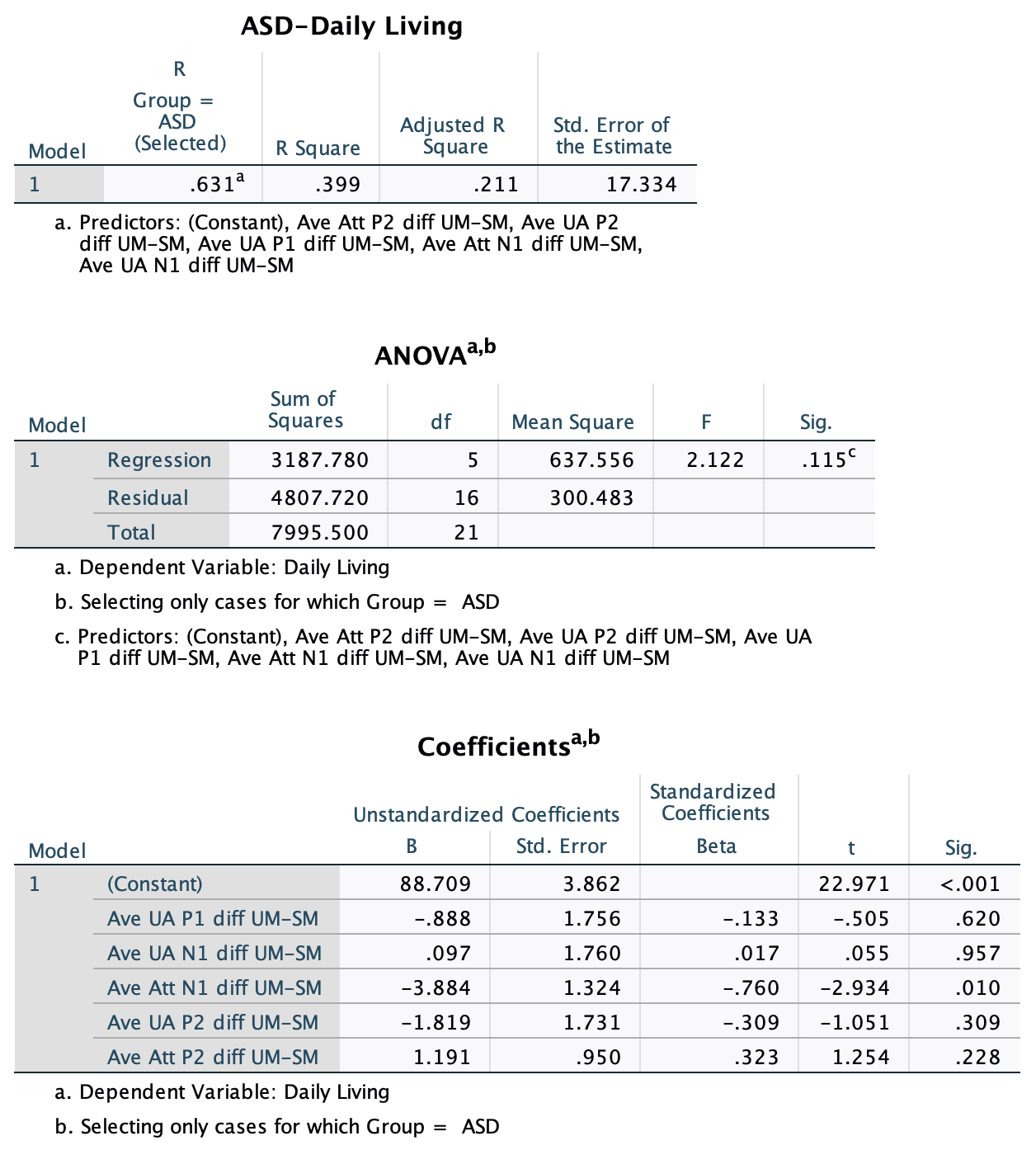


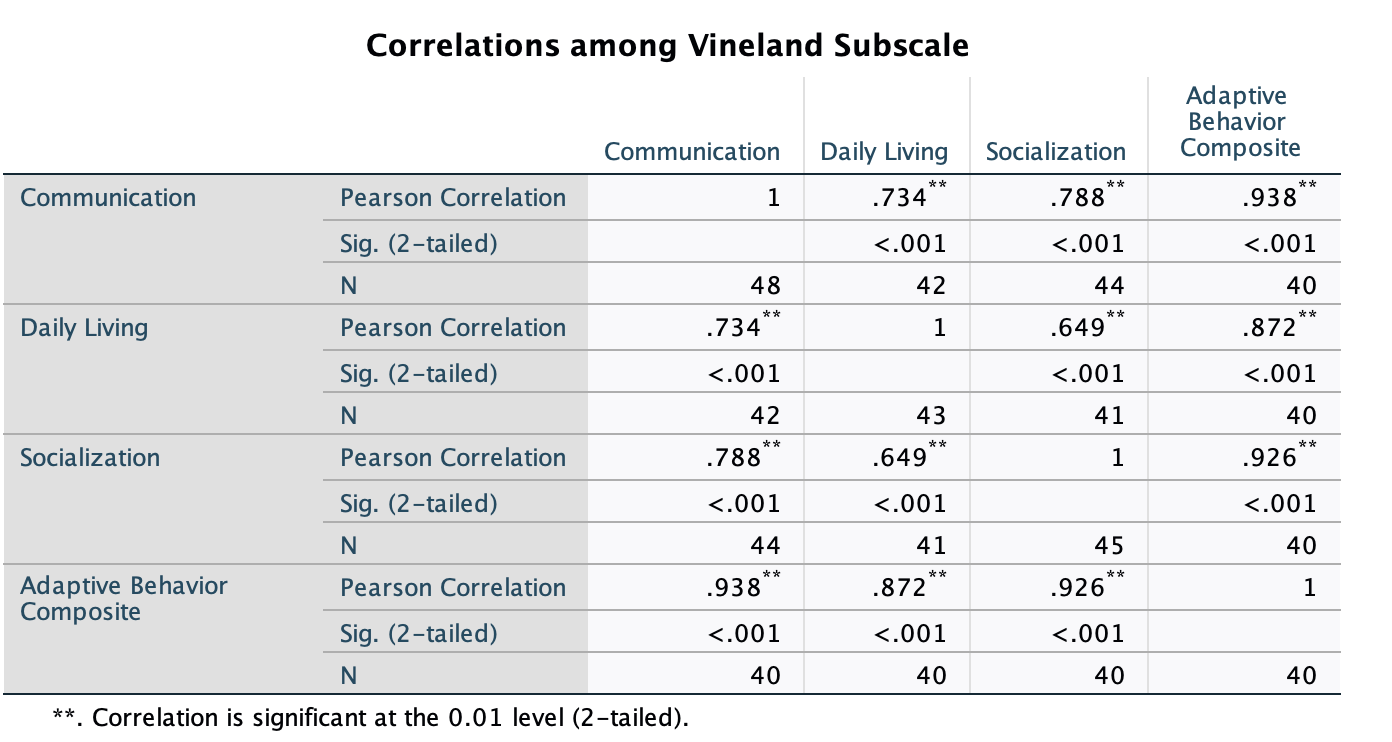

Supplement: Supplementary file 8 — Additional file 8: EEG–phenotype correlations (additional Vineland subscales). [file 13229_2022_512_MOESM8_ESM.docx]
